# Differential control of Zap1-regulated genes in response to zinc deficiency in *Saccharomyces cerevisiae*

**DOI:** 10.1186/1471-2164-9-370

**Published:** 2008-08-01

**Authors:** Chang-Yi Wu, Amanda J Bird, Lisa M Chung, Michael A Newton, Dennis R Winge, David J Eide

**Affiliations:** 1 Department of Nutritional Sciences, University of Wisconsin-Madison, Madison, WI 53706, USA; 2 Department of Biochemistry, University of Utah, Salt Lake City, UT 84132, USA; 3 Department of Statistics, University of Wisconsin-Madison, Madison, WI 53706, USA

## Abstract

**Background:**

The Zap1 transcription factor is a central player in the response of yeast to changes in zinc status. We previously used transcriptome profiling with DNA microarrays to identify 46 potential Zap1 target genes in the yeast genome. In this new study, we used complementary methods to identify additional Zap1 target genes.

**Results:**

With alternative growth conditions for the microarray experiments and a more sensitive motif identification algorithm, we identified 31 new potential targets of Zap1 activation. Moreover, an analysis of the response of Zap1 target genes to a range of zinc concentrations and to zinc withdrawal over time demonstrated that these genes respond differently to zinc deficiency. Some genes are induced under mild zinc deficiency and act as a first line of defense against this stress. First-line defense genes serve to maintain zinc homeostasis by increasing zinc uptake, and by mobilizing and conserving intracellular zinc pools. Other genes respond only to severe zinc limitation and act as a second line of defense. These second-line defense genes allow cells to adapt to conditions of zinc deficiency and include genes involved in maintaining secretory pathway and cell wall function, and stress responses.

**Conclusion:**

We have identified several new targets of Zap1-mediated regulation. Furthermore, our results indicate that through the differential regulation of its target genes, Zap1 prioritizes mechanisms of zinc homeostasis and adaptive responses to zinc deficiency.

## Background

Organisms require mechanisms to survive under adverse conditions of extreme heat, osmolarity, nutrient limitation, and other stresses. Recent studies have begun to probe the cellular responses to the stress of zinc deficiency. Zinc is a critical cofactor for many proteins and plays important roles in myriad biological processes. Therefore, when zinc becomes limiting, cells must respond to maintain zinc homeostasis. In addition, cells may alter their metabolic processes to adapt to growth under conditions where certain zinc-dependent proteins are less active. We are examining the cellular responses to zinc deficiency in the yeast *Saccharomyces cerevisiae*.

In this yeast, the Zap1 transcription factor is a central player in the response to zinc deficiency [1]. For many of its target genes, Zap1 acts as an activator of transcription and increases gene expression when zinc levels are low. To perform this function, Zap1 binds to Zinc-Responsive Elements or "ZREs" in the promoters of its target genes [2]. The consensus sequence for a ZRE is ACCTTNAAGGT. While some Zap1 target genes contain multiple functional ZREs, many others have only a single binding site [2,3].

The Zap1 protein is 880 amino acids long. A DNA binding domain consisting of five zinc fingers is found at its carboxy terminus [4,5]. In addition, Zap1 contains two independent activation domains, designated AD1 and AD2, that mediate the increased transcription of target genes [6]. Zap1 is a direct sensor of cellular zinc levels. The protein resides in the nucleus under all conditions of zinc status [6]. When zinc levels rise, the metal binds to ligand residues in the AD1 and AD2 regions of the protein and this binding inhibits the ability of these domains to promote transcription [6-10]. Alteration of these regulatory zinc-binding ligands by mutation decreases the ability of Zap1 to respond to zinc and the mutant protein constitutively activates transcription [7,10].

Previous studies have identified a large number of potential Zap1 target genes in the yeast genome [3,11,12]. Many of these genes act to maintain sufficient levels of cytosolic zinc available for cell growth. For example, the *ZRT1*, *ZRT2*, and *FET4 *genes encode zinc transporters responsible for zinc uptake across the plasma membrane [13-15]. These genes are induced by Zap1 in zinc-limited cells. Zap1 also induces expression of the *ZRT3 *gene in low zinc; *ZRT3 *encodes a vacuolar membrane protein responsible for transporting zinc stored in the vacuole to the cytoplasm for its utilization [16]. As a final example, Zap1 induces transcription of its own gene in a positive autoregulatory loop [1]. Thus, Zap1 levels rise in zinc-limited cells and this may lead to increased expression of other target genes.

In addition to its role in activating gene expression, Zap1 can also act as a transcriptional repressor. Previous studies have identified two different mechanisms of Zap1-mediated repression. The *ZRT2 *gene provided the first example. *ZRT2 *is unusual among Zap1 target genes in that it is induced by mild zinc limitation and repressed by more severe zinc deficiency [17]. This paradoxical pattern of regulation is due to the presence of three ZREs in the *ZRT2 *promoter. Two high affinity ZREs, ZRE1 and ZRE2, are located upstream of the TATA box and these elements mediate Zap1-dependent activation of gene expression. The third ZRE, ZRE3, has a low affinity of Zap1 binding and is located downstream of the TATA box. ZRE3 is essential for repression of *ZRT2 *expression. Under mild conditions of zinc deficiency, Zap1 binds to ZRE1 and ZRE2 and activates gene expression. Under severe zinc deficiency, Zap1 levels rise due to autoregulation and the protein then binds to ZRE3 and interferes with *ZRT2 *expression possibly by blocking transcription initiation.

The *ADH1 *and *ADH3 *genes provide examples of a second mechanism of Zap1-mediated repression. *ADH1 *and *ADH3 *encode zinc-dependent alcohol dehydrogenases. These genes are highly expressed in zinc-replete cells but are repressed in zinc-deficient cells [18]. Zap1 mediates *ADH1 *and *ADH3 *repression in low zinc by means of intergenic transcripts that are activated by Zap1 and transcribed through the *ADH1 *and *ADH3 *promoters. These intergenic transcripts, designated *ZRR1 *and *ZRR2 *respectively, do not encode protein products but rather their synthesis results in the transient displacement of transcription factors normally required for *ADH1 *and *ADH3 *expression. This results in the reduced expression of two of the most abundant zinc-binding proteins in the cell. Conversely, the *ADH4 *gene is induced by Zap1 and this gene encodes a potential iron-dependent alcohol dehydrogenase [3,18,19]. By switching from zinc-dependent to zinc-independent ADH isozymes, the cell may conserve zinc for other uses. Alternatively, Adh4 may use zinc as its cofactor [20] but this protein is predicted to bind only one zinc per monomer while Adh1 and Adh3 each bind two. Thus, zinc conservation could occur under this scenario as well.

DNA microarrays have been remarkably useful in assessing the transcriptional responses of an organism such as yeast to stress conditions [3,11,12]. In a previous study, we used microarrays to identify likely Zap1 target genes in the yeast genome, a group of genes that we referred to as the Zap1 "regulon" [3]. In that study, we identified a total of 46 genes in yeast that are potential targets of Zap1 activation. As described below, we have further addressed this issue using revised experimental approaches. This new analysis has led to the identification of many new potential Zap1 target genes. In addition, we have characterized the differential regulation of Zap1 target genes. We found that some genes respond to mild zinc deficiency and act as a first line of defense against this stress. These first-line defense genes participate in various mechanisms of zinc homeostasis. Other Zap1 target genes respond only to severe conditions of zinc limitation and serve as a second line of defense. Second-line defense genes act largely in the adaptation to conditions where zinc availability is insufficient to maintain optimal cell function.

## Results

### Identification of new Zap1 target genes

Our previous study suggested that Zap1 may directly activate the expression of as many as 46 different genes [3]. That study used genome-wide transcription profiling with DNA microarrays coupled with a motif identification algorithm, MEME. Specifically, we first compared gene expression in wild type cells grown in low and high zinc and identified 458 genes expressed more highly in low zinc in duplicate microarrays. We refer to this as "Experiment 1" or "E1." We then compared gene expression in wild type and *zap1Δ *mutant cells grown in low zinc and identified 214 genes that were expressed at a higher level in wild type cells in duplicate arrays. We refer to this condition as "Experiment 2" or "E2." A set of 111 genes showed increased expression in both E1 and E2. MEME identified potential ZRE sequences located within the promoter regions of 46 of the 111 genes. These results suggested that these 46 genes are direct targets of Zap1 gene regulation.

**Figure 1:**
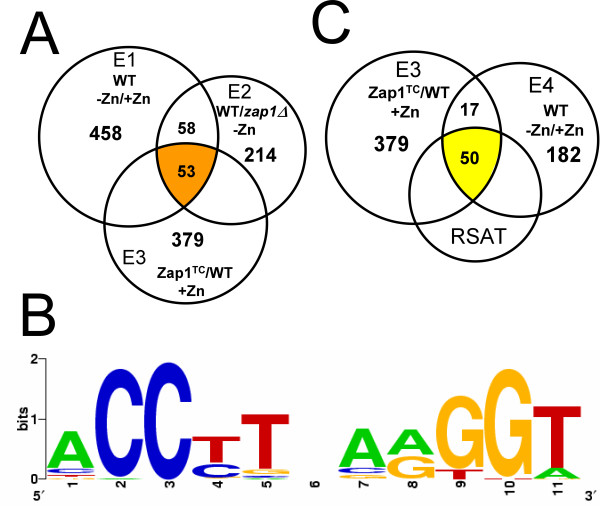
**New strategies to identify genes regulated by Zap1.** A) Identifying genes affected by moderate zinc deficiency. Experiments E1, E2, and E3 were combined to identify genes that showed increased expression in zinc-limited cells (E1), wild type cells vs. *zap1Δ *mutant cells in low zinc (E2), and Zap1^TC^-expressing cells in high zinc (E3). B) The ZRE sequences in the promoters of the previously identified 46 Zap1 target genes [3] were aligned and a logo was built using WebLogo. C) Experiments E3 and E4 were combined to identify Zap1 targets that respond to severe zinc deficiency. Regulatory Sequence Analysis Tools (RSAT) was used to identify potential ZREs in the promoters of co-regulated genes.

In this current study, we have further characterized the Zap1 regulon using additional microarray experiments and a more sensitive motif identification algorithm. First, we devised a third microarray experiment to identify Zap1 target genes. Since our previous analysis, we have generated alleles of Zap1 that are constitutive and poorly regulated by zinc. One such allele, designated "Zap1^TC^", was used in this study. The Zap1^TC ^allele contains mutations in or near the two activation domains of Zap1 that render those domains less responsive to zinc [7]. We predicted that Zap1 target genes would show increased expression in zinc-replete cells overexpressing the Zap1^TC ^allele. Microarray experiments were performed in which wild-type cells expressing the Zap1^TC ^allele and wild-type cells bearing the vector only were grown in high zinc conditions. We refer to this as "Experiment 3" or "E3." Expression of 379 genes increased in Zap1^TC^-expressing cells an average of ≥ 1.5-fold in duplicate arrays. When combined with our previous results, a total of 53 genes were found to have the expected alterations of expression in E1, E2, and E3 conditions (Fig. 1A).

While MEME was very useful for our previous analysis, we became concerned about the ability of this algorithm to identify functional ZREs. For example, MEME was unable to identify experimentally confirmed ZREs in the promoters of the *ZRT2*, *PIS1*, and *EKI1 *genes [17,21,22] (data not shown). These observations suggested that MEME was not sufficiently sensitive to identify all potential ZREs within a collection of target gene promoters. Therefore, we analyzed the promoters of the 53 candidate genes (Fig. 1A) using a more sensitive motif identification algorithm called Regulatory Sequence Analysis Tools or RSAT. With RSAT, a position-specific probability matrix was generated using the ZREs identified by MEME and a representation of that consensus sequence is provided in Fig. 1B. This matrix was then used to search for additional ZRE-like sequences in the promoters of these genes. Sequences were scored based on their similarity to the probability matrix with higher scores indicating greater similarity. An RSAT score of 4.5 was used as a minimum cut-off value in this analysis because this is the lowest score obtained for a bona fide ZRE found in the *EKI1 *promoter [21]. Our reasoning was that sequences scoring ≥ 4.5 were strong candidates for functional ZREs. Among the 53 genes implicated to be Zap1 targets by the combination of E1, E2, and E3, the promoters of 49 genes contain one or more potential ZREs identified by RSAT and these are listed in Tables 1 and 2. Table 1 lists 33 genes that were identified as potential Zap1 targets in our previous experiments. While several of these have been demonstrated to be Zap1 targets by additional experiments (*DPP1*, *IZH1*, etc), several others (*MOH1*, *TKL2*, etc.) had not been further characterized since our initial analysis. For all genes listed, the new E3 analysis provided additional experimental support that these genes are indeed Zap1 targets.

**Table 1:** Confirmation of previously identified Zap1 target genes by E1, E2, and E3 clustering.

**ORF^a^**	**Gene**	**Function**	**Fold induction**				**ZRE**		
				
			**E1^b^**	**E2^b^**	**E3-1^c^**	**E3-2^c^**	**start^d^**	**score^e^**	**sequence**
**Experimentally tested Zap1 target genes**									

YDR284C	*DPP1*	diacylglycerol pyrophosphate phosphatase	5.5	3.6	2.4	2.2	-452	9.6	ACCTGAAAGGT
YDR492W	*IZH1*	membrane steroid hormone receptor ortholog	2.8	4.4	1.4	1.6	-914	5.0	TCCCTCAATGA
							-417	11.2	ACCCTAAAGGT
YGL255W	*ZRT1*	plasma membrane zinc uptake transporter	24.2	18.9	38.8	34.4	-456	9.3	ACCTTTGGGGT
							-338	7.7	ACCTCGAAGGA
							-319	12.0	ACCTTGAGGGT
							-204	11.7	ACCTTGAAGGT
YGL256W	*ADH4*	alcohol dehydrogenase IV	26.2	5.6	12.6	12.6	-269	9.4	ACCGTGAAGGT
							-191	5.3	GCCTTCAATGA
YJL056C	*ZAP1*	zinc-responsive transcriptional activator protein	7.5	16.9	96.5	79.7	-144	11.2	ACCCTAAAGGT
YKL175W	*ZRT3*	vacuolar zinc export transporter	7.9	8.7	4.9	4.8	-155	11.6	ACCTTAAGGGT
YLR130C	*ZRT2*	plasma membrane zinc uptake transporter	5.6	12.3	2.7	2.5	-941	6.1	ACCCTAACTGT
							-311	11.2	ACCCTAAAGGT
							-262	11.2	ACCCTAAAGGT
							-174	5.8	ACCTTTTGGGA
							-112	4.9	ACCAACAGGGT
							-41	5.3	GCCTGCAATGT
YMR243C	*ZRC1*	vacuolar zinc import transporter	2.1	4.7	1.6	1.8	-174	9.6	GCCTTGAAGGT
YMR319C	*FET4*	plasma membrane Fe/Cu/Zn uptake transporter	2.3	2.4	1.6	1.7	-384	7.8	ACCCCACGGGT
YNR039C	*ZRG17*	endoplasmic reticulum zinc import transporter	3.2	3.5	2.3	1.9	-527	11.7	ACCTTGAAGGT
YOL002C	*IZH2*	membrane steroid hormone receptor ortholog	1.9	2.0	3.4	3.3	-256	7.7	ACCCTAGAGGA
							-173	7.2	ACCCCGAGTGT
YOL154W	*ZPS1*	cell wall protein, putative metalloprotease	13.9	10.1	78.6	57.9	-329	11.8	ACCTTCAGGGT
							-314	11.8	ACCTTCAGGGT

**Likely Zap1 target genes**									

YBL049W	*MOH1*	zinc-binding protein, function unknown	10.7	2.2	3.2	3.8	-387	8.7	CCCTTGAGGGA
YBR117C*	TKL2*	transketolase, pentose phosphate pathway	5.4	2.0	1.9	2.1	-844	8.2	ACCTTATGGGT
							-524	5.3	ACCCAAAATGT
YDR055W*	PST1*	cell wall protein	5.0	2.7	2.0	1.9	-886	7.4	ACCCCAAGGGA
							-405	8.0	TCCTTGAGGGA
YGL121C	*GPG1*	putative gamma subunit of a heterotrimeric G protein	10.8	2.7	2.1	2.4	-287	8.3	CCCTTCGAGGT
							-208	9.0	ACCGTAAAGGT
YGL257C	*MNT2*	mannosyltransferase, O-linked glycosylation	2.3	4.4	4.0	3.7	-572	5.3	GCCTTCAATGA
							-494	9.4	ACCGTGAAGGT
YGL258W	*VEL1*	function unknown	16.3	4.0	112.6	78.6	-763	6.3	ACCTTGCATGA
							-232	10.4	ACCCTGCGGGT
YGR295C	*COS6*	function unknown	2.7	2.2	1.5	1.6	-875	5.0	AACTTAAATGT
							-309	9.1	ACCTTAAATGT
YJL132W*		similar to phospholipase D	5.2	3.8	3.3	2.8	-154	7.8	ACCCAAAGGGT
YJR061W		Mnn4-related, N-linked glycosylation	4.6	2.1	2.0	1.8	-278	9.8	ACCTTCAAGGA
YKL165C	*MCD4*	glycosylphosphatidylinositol (GPI) anchor synthesis	4.1	2.5	1.8	1.8	-104	11.4	ACCTTAAAGGT
YLL020C		function unknown	5.1	3.1	1.7	1.6	-933	12.0	ACCTTGAGGGT
YMR120C	*ADE17*	AICAR transformylase, purine biosynthesis	5.2	3.7	1.9	2.2	-98	8.7	ACCTTTAGTGT
YMR271C	*URA10*	orotate phosphoribosyltransferase, pyrimidine biosynthesis	6.1	2.5	4.9	5.2	-143	7.9	ACCTTTCGGGA
YMR297W	*PRC1*	vacuolar carboxypeptidase Y	3.0	3.5	1.5	1.9	-182	8.1	ACCCCGCGGGT
YNL254C		function unknown	9.7	12.0	2.3	2.0	-432	11.7	ACCTTGAAGGT
YNL336W	*COS1*	function unknown	2.1	2.0	1.6	1.6	-871	5.0	AACTTAAATGT
							-591	5.3	AACCTAGAGGT
							-314	9.1	ACCTTAAATGT
YOL084W	*PHM7*	major facilitator superfamily member, function unknown	9.7	2.1	2.5	2.3	-766	11.7	ACCTTGAAGGT
YOL131W		function unknown	5.0	2.3	2.5	2.2	-1000	7.6	AACTTCAGGGT
							-458	8.2	ACCTGGAAGGA
							-364	5.9	ACCCAAGAGGT
							-122	5.5	TCCATTAGGGT
YOR387C		function unknown	17.8	19.3	136.4	113.6	-763	6.3	ACCTTGCATGA
							-231	10.4	ACCCTGCGGGT
YOR134W	*BAG7*	GTPase-activating protein, control of cell wall synthesis	5.9	2.0	1.7	1.6	-445	6.0	CCCTCCAAGGA
							-325	8.6	CCCCTGCAGGT
YPL250C*	ICY2*	function unknown	2.1	2.3	3.1	3.1	-756	8.9	CCCTTCCGGGT
							-524	7.4	ACCCCAAGGGA
							-475	5.7	GCCCAAAGGGT
							-360	5.6	CCCGTCAGTGT

**Figure 2:**
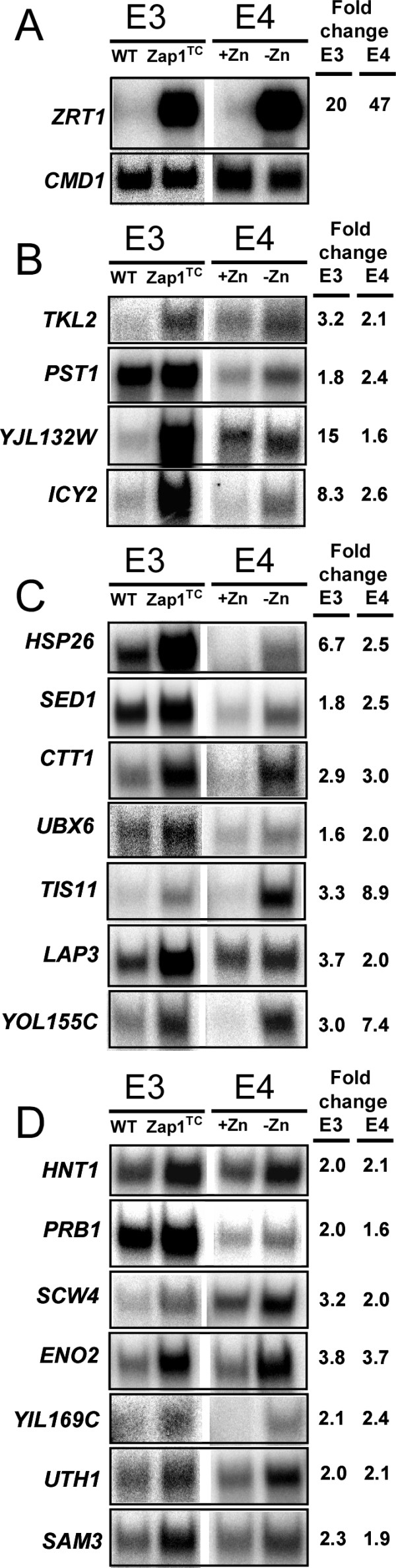
**Confirmation of the microarray results for potential Zap1 target genes.** S1 nuclease protection assays were performed using RNA isolated from cells grown under the same conditions as microarray experiments E3 and E4. A) *ZRT1 *and *CMD1 *were used as positive and loading controls, respectively. Results with candidate genes from Table 1 (B), Table 2 (C) and Table 3 (D) are shown. The band intensities were quantified and normalized to *CMD1 *levels, and the fold increase in E3 and E4 conditions is reported. These data confirmed the microarray results for these genes.

a) Results for genes marked with asterisks were confirmed independently by S1 nuclease protection assay in Figure 2.

b) Expression ratios are the average of two independent microarray experiments (Lyons et. al. 2000).

c) Results from two independent microarray experiments (E3-1 and E3-2) are shown.

d) Numbers indicate the distance from the ATG initiation codon.

e) Score calculated for each sequence with a position-specific scoring matrix generated by RSAT.

**Table 2:** New candidate Zap1 target genes identified by E1, E2, and E3 clustering.

**ORF^a^**	**Gene**	**Function**	**Fold induction**				**ZRE**		
				
			**E1^b^**	**E2^b^**	**E3-1^c^**	**E3-2^c^**	**start^d^**	**score^e^**	**sequence^f^**
YBL029W		function unknown	3.0	2.9	1.6	1.4	-995	5.8	CCCCTGCCGGT
YBR072W*	HSP26*	small heat shock protein, protein folding	40.5	2.0	3.3	3.5	-451	5.3	**ACCTTGCCTGT**
YDR077W*	SED1*	cell wall protein	2.6	2.2	2.6	2.1	-896	4.7	**CCCTTATAGGA**
YGR088W*	CTT1*	catalase T, oxidative stress resistance	17.1	3.7	1.9	1.7	-337	5.7	CCCTTACCGGT
YGR243W	*FMP43*	mitochondrial protein, function unknown	2.8	2.7	3.1	2.5	-552	4.9	TCCTCGAATGT
YHR214W-A		function unknown	6.5	2.6	3.0	3.2	-274	4.8	ACCTCTGGTGT
YIL045W	*PIG2*	putative protein phosphatase regulatory subunit	4.6	2.4	1.6	1.5	-859	4.7	ACCTTCCACGT
YJL048C*	UBX6*	UBX (ubiquitin regulatory X) domain-containing protein	2.3	3.3	1.8	1.6	-198	5.2	**TCCATTAAGGT**
							-39	5.9	CCCTCAAAGGA
YKR046C	*PET10*	lipid particle protein, unknown function	2.9	3.0	1.7	1.7	-929	4.8	ATCTTGCAGGT
YLR136C*	TIS11*	tristetraproline homolog, control of mRNA stability	2.6	4.1	5.6	3.7	-821	4.9	**GCCCGTGAGGT**
							-768	6.3	AACCTGCGGGT
							-507	6.0	GCCCAGAGGGT
YML028W	*TSA1*	peroxiredoxin, oxidative stress resistance	5.0	2.9	2.0	1.5	-195	5.5	**GCCCGTCGGGT**
							-170	7.2	TCCCTAAAGGA
YMR181C		function unknown	5.9	2.2	2.6	2.7	-237	5.1	CCCTTCGAGGG
YNL239W*	LAP3*	homocysteine thiolactonase	2.1	4.7	2.9	2.9	-947	4.8	GCCTCCAATGT
YOL082W	*ATG19*	autophagy and cytoplasm-to-vacuole (CVT) targeting pathway	4.3	3.2	1.6	1.4	-281	5.9	ACCTTAAAAGT
							-998	4.9	ACCAACAGGGT
YOL155C*		cell wall protein	11.8	3.5	2.3	2.9	-361	6.3	**ACCGTGCAGGA**
YPL159C	*PET20*	mitochondrial protein required for respiratory growth	9.1	5.2	4.5	4.0	-278	5.3	GCCATTAAGGT
							-180	5.6	ACCCTTTGGGA

**Figure 3:**
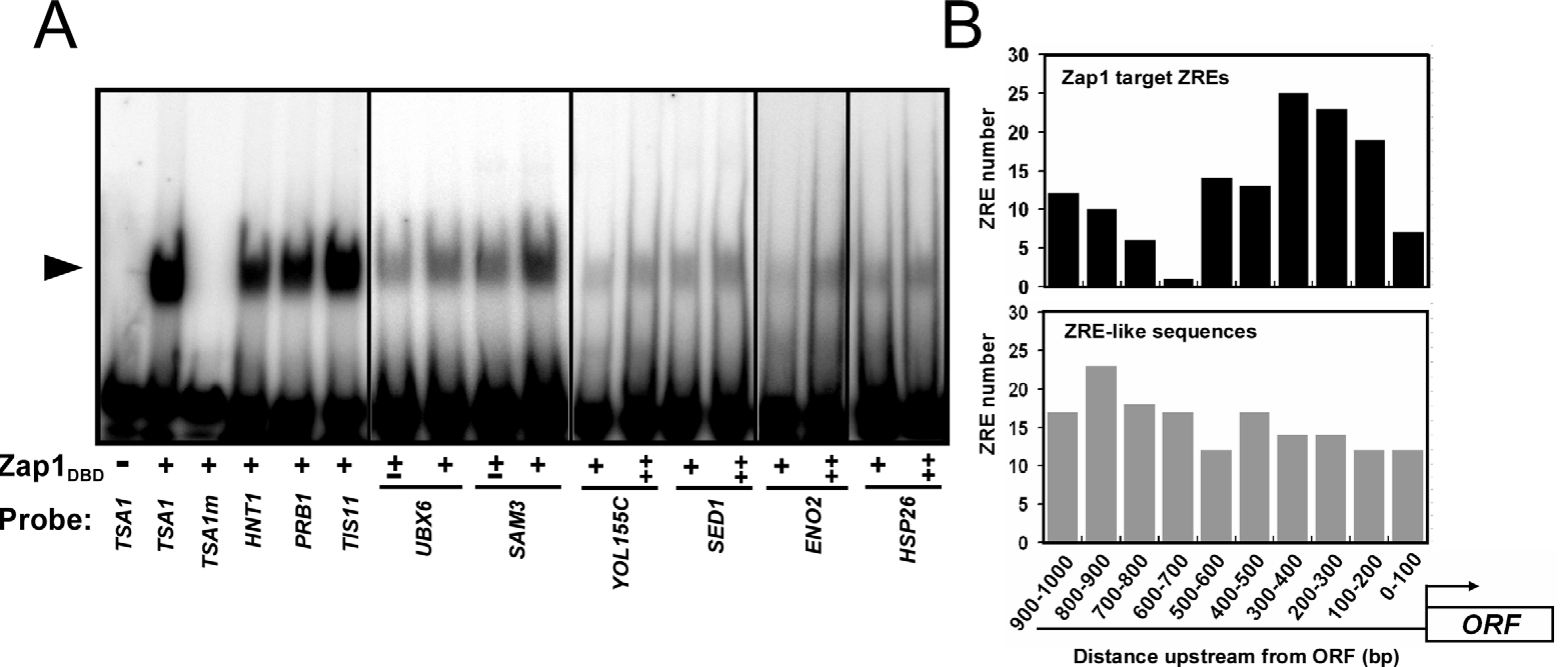
**Evidence that ZRE sequences identified by RSAT are functional Zap1 binding sites.** A) Electrophoretic mobility shift assay of candidate ZREs. Radiolabeled double-stranded oligonucleotides (0.5 pmol, 10,000 cpm) containing potential ZRE sequences from the indicated promoters were used as probes. The probes were mixed with 0 (-), 0.2 (±), 0.4 (+), or 0.8 (‡) μg per reaction of purified Zap1 DNA binding domain (Zap1_DBD_). The *arrow *indicates the Zap1_DBD_-DNA complex. The bona fide ZRE from *TSA1 *was used as a positive control and a mutant nonfunctional allele of that sequence (TSA1m) was used as a negative control. B) Nonrandom distribution of ZRE-like sequences in candidate Zap1 target gene promoters. In the *upper *panel, the positions of ZRE-like sequences in candidate Zap1 target promoters are plotted relative to the distance from the ATG start codon of the corresponding ORF. In the *lower *panel, the positions of ZRE-like sequences identified in the promoters of genes not showing zinc- and/or Zap1-responsive gene expression are plotted.

a) Results for genes marked with asterisks were confirmed independently by S1 nuclease protection assay in Figure 2.

b) Expression ratios are the average of two independent microarray experiments (Lyons et. al. 2000).

c) Results from two independent microarray experiments (E3-1 and E3-2) are shown.

d) Numbers indicate the distance from the ATG initiation codon.

e) Score calculated for each sequence with a position-specific scoring matrix generated by RSAT.

f) ZREs shown in bold were tested for interaction with Zap1 as described in Figure 3A.

Table 2 lists 16 new candidate Zap1 target genes identified in this study. These genes were not identified in our previous analysis due to the insensitivity of MEME in detecting ZRE-like sequences. This group includes *HSP26*, *SED1*, *CTT1*, and *TSA1*.

We noted that 13 of the 46 Zap1 target genes previously identified were not clearly up-regulated in cells expressing the Zap1^TC ^allele (E3). The E1, E2, and E3 results for these genes are provided in Additional file 1. While several of these genes showed increased expression in the Zap1^TC^-expressing cells, these increases did not satisfy our cut-off criteria. It is unclear at this time why these genes were less responsive to the constitutive Zap1 allele than other targets. We also found that 4 genes (*ALD2*, *PIR3*, *YBR285W*, *YNR066C*) showed increased expression in E1, E2, and E3 but did not contain ZREs in their promoters that were detectable by RSAT (Additional file 2). These genes may contain ZRE sequences that are more divergent from the consensus. Alternatively, Zap1 may alter their expression indirectly. It should be noted that *ALD2 *is closely related to *ALD3*, which was found to be a potential Zap1 target (Table 3). Therefore, *ALD2 *may have been detected due to cross-hybridization with *ALD3 *mRNA in the microarray experiments.

**Table 3:** New candidate Zap1 target genes identified by E3 and E4 clustering.

**ORF^a^**	**Gene**	**Function**	**Fold induction^b^**				**ZRE**		
				
			**E3-1**	**E3-2**	**E4-1**	**E4-2**	**start^c^**	**score^d^**	**sequence^e^**
YDL125C*	HNT1*	adenosine 5'-monophosphoramidase	1.9	1.6	3.6	4.1	-279	6.9	**GCCTCAAAGGT**
YEL060C*	PRB1*	vacuolar proteinase B	2.6	2.8	2.0	2.1	-677	6.5	**GCCATGAGGGT**
YFL014W	*HSP12*	cell wall protein, stress resistance	1.5	1.5	4.4	3.7	-211	5.0	ACCTCAAAGTT
YGR254W	*ENO1*	enolase I, glycolysis and gluconeogenesis	2.6	2.9	1.4	1.7	-927	9.4	ACCGTGAAGGT
							-371	5.1	ACCTGAGCGGT
							-216	5.6	CACCTCAAGGT
YGR279C*	SCW4*	cell wall protein with similarity to glucanases	1.8	1.9	1.5	1.4	-481	5.4	CCCTGCACGGT
							-466	6.3	ACCCTCTGGGA
YHR174W*	ENO2*	enolase II, glycolysis and gluconeogenesis	2.5	2.8	1.3	1.6	-594	4.8	**ACGCTGCGGGT**
YIL169C*		cell wall potein	3.4	3.1	2.1	2.2	-742	10.8	ACCCGGAAGGT
							-252	6.0	ACCTCGCAGGC
YJL052W	*TDH1*	glyceraldehyde-3-phosphate dehydrogenase	1.4	1.6	1.6	1.6	-347	4.6	ACCTTCGGAGT
YJL171C		cell wall protein	1.6	1.8	1.6	1.8	-573	4.9	CCCATAAAGGA
YKR042W*	UTH1*	mitochondrial protein, oxidative stress resistance	3.5	4.0	1.4	1.5	-282	6.9	CCCTTCAATGT
YLL053C		aquaporin	1.6	1.5	1.7	1.5	-354	5.5	ACCGGTCGGGT
YMR169C	*ALD3*	aldehyde dehydrogenase	2.0	1.9	1.6	1.5	-214	9.1	TCCCTAAGGGT
							-79	4.6	ACCTGGCATGA
YOR348C	*PUT4*	proline permease	1.8	1.8	1.9	1.8	-299	8.2	CCCTGCAAGGT
YPL274W*	SAM3*	S-adenosylmethionine permease	1.9	1.7	2.1	2.2	-240	4.7	**TCCCCTGCGGT**
YPR003C		putative ER sulfate permease	2.2	2.1	1.5	1.5	-149	5.5	ACCGAAAAGGT

a) Results for genes marked with asterisks were confirmed independently by S1 nuclease protection assay in Figure 2.

b) Results from two independent microarray experiments (E3-1, E3-2; E4-1, E4-2) for each condition are shown.

c) Numbers indicate the distance from the ATG initiation codon.

d) Score calculated for each sequence with a position-specific scoring matrix generated by RSAT.

e) ZREs shown in bold were tested for interaction with Zap1 as described in Figure 3A.

### Identification of Zap1 targets induced by severe zinc deficiency

In our previous study, cells grown in the low zinc conditions used in E1 and E2 experiments were cultured in CSD medium from which zinc was removed with a metal-binding resin [3]. This medium was chosen to specifically provide a zinc-limiting condition without the use of strong chelators that can bind other metal ions and alter their availability. However, because chelators are not included in CSD, this medium is not severely zinc limiting due to the presence of small amounts of contaminating zinc. We reasoned that some Zap1 targets might require extremely low zinc conditions, i.e. lower than that provided by CSD, to be induced. Therefore, to search for additional Zap1 target genes, microarray experiments were performed with RNA from cells grown in LZM, a low zinc medium containing EDTA and citrate as metal buffers. Because of these chelators, cells grown in LZM + 3 μM added ZnCl_2 _are more zinc-limited than cells grown in CSD with no added zinc. When comparing expression in cells grown in LZM + 3 μM ZnCl_2 _vs. LZM + 3 mM ZnCl_2_, 182 genes were up-regulated ≥ 1.5-fold in duplicate arrays under this very low zinc condition. We refer to this experiment as "E4." The overlap between E3 and E4 was 67 genes (Fig. 1C). Of these 67 genes, 50 contained ZRE-like sequences in their promoters detectable by RSAT. Fifteen of these 50 genes were not detected in the more mild E1/E2 growth conditions suggesting that they are only induced under severe zinc-limiting conditions. A list of these additional potential Zap1 target genes is provided in Table 3.

To summarize this analysis, we have obtained additional evidence that 33 genes previously identified in our experiments are Zap1 targets (Table 1). In addition, we have identified a total of 31 new candidate Zap1 target genes (Tables 2 and 3). The functions of many of these genes and their possible relevance to zinc deficiency will be discussed later in this report.

### Confirmation of microarray and RSAT results

To confirm the effects of zinc status and Zap1 mutations on potential target gene expression, a subset of genes from Tables 1, 2, 3 were selected for further analysis by S1 nuclease protection assay of RNA samples that were not used for the microarray analysis. The known Zap1 target *ZRT1 *and the constitutive calmodulin (*CMD1*) gene were used as positive and negative controls, respectively (Fig. 2A). Four genes from Table 1 previously suggested to be Zap1 targets (*TKL2, PST1*, *YJL132W*, *ICY2*) were tested using cells grown under the same conditions as were used for microarray experiments E3 and E4. The increased expression of these genes in cells grown in low zinc or in cells expressing the Zap1^TC ^allele was confirmed by this analysis (Fig. 2B). Similarly, we tested 14 of the 31 new candidate Zap1 target genes, i.e. 7 from Table 2 (Fig. 2C) and 7 from Table 3 (Fig. 2D). The expression patterns of all 14 of these genes were confirmed by S1 nuclease protection assay. These data indicate that we have discovered several new genes directly regulated by Zap1.

The RSAT algorithm identified several potential ZREs in addition to those recognized by MEME. Many of these sequences had low RSAT scores near the minimum cut-off value of 4.5 and were very divergent from the consensus ZRE sequence (ACCTTNAAGGT, RSAT score = 11.7) (Fig. 1B). To determine if Zap1 can bind to these candidate ZREs in a sequence-specific manner, we performed electrophoretic mobility shift assays (EMSA) (Fig. 3A). The Zap1 DNA binding domain (Zap1_DBD_, residues 687–880) was purified from *E. coli *and used in these *in vitro *experiments. Double-stranded oligonucleotides were end-labeled with ^32^P, mixed with the purified Zap1_DBD_, and then fractionated by non-denaturing polyacrylamide gel electrophoresis. Binding of Zap1_DBD _to the previously characterized *TSA1 *ZRE (RSAT score = 5.5) served as a positive control (Fig. 3A, lane 2). Zap1_DBD _was unable to bind to a *TSA1 *ZRE that had been mutagenized at each of its 11 base pair positions by transversion mutations (Fig. 3A, lane 3). This mutant ZRE was previously shown to be nonfunctional *in vivo *[23]. Zap1_DBD _binding was detected with all of the candidate ZREs tested. These sequences ranged in RSAT scores from a low of 4.7 (*SAM3*, *SED1*) to a high of 6.9 (*HNT1*). The ability of Zap1_DBD _to bind to these sequences *in vitro *suggests that they may be functional binding sites *in vivo*.

Additional evidence that the identified ZREs are functional *in vivo *came from an analysis of the distribution of these sequences within the promoter regions of candidate Zap1 target genes. As shown in Fig. 3B, when the position of the RSAT-predicted ZREs were mapped relative to the open reading frame of each gene, a clear bias was apparent; most of these sequences were found between 100 and 500 base pairs upstream. This distribution correlates well with the position of regulatory elements in other yeast promoters [24]. Chi-square analysis indicated that this distribution of ZRE sequences was not random (P < 0.001). As an additional control, we identified 156 ZRE-like sequences in the promoters of yeast genes that do not show Zap1-dependent regulation and are therefore unlikely to be functional Zap1 binding sites (see Materials and Methods). The locations of these nonfunctional sequences were consistent with a random pattern of distribution (P > 0.05). Taken together, these results suggest that RSAT is a sensitive method to detect regulatory motifs among the promoters of co-regulated genes that are likely to be functional.

### Differential regulation of Zap1 target genes

**Figure 4:**
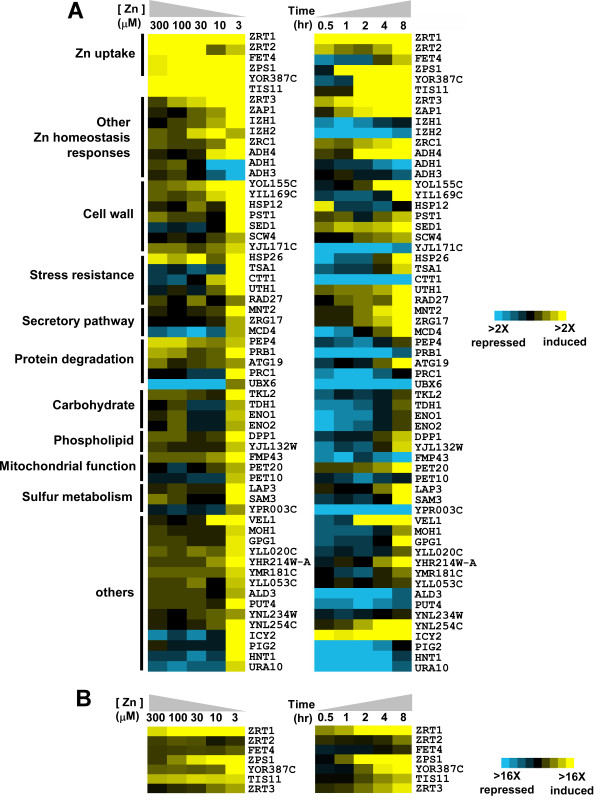
**Differential regulation of Zap1 target genes in response to zinc.** A) Microarray studies were performed with cells grown over a range of zinc (*left *panel) or over time after zinc withdrawal (*right *panel). For the dose-response analysis, cells were grown in LZM + 3 mM ZnCl_2_, or LZM + 300, 100, 30, 10 or 3 μM ZnCl_2_. Transcript levels were assayed using microarrays in which each sample was paired with the zinc-replete (LZM + 3 mM ZnCl_2_) control. For the time-course studies, cells were grown to exponential phase in a zinc-replete medium (LZM + 1 mM ZnCl_2_) and then transferred to a zinc-limiting medium (LZM + 1 μM ZnCl_2_) for 8 hours. RNA was isolated at the indicated times and transcript levels were assayed using microarrays in which each sample was paired with the zinc-replete T_o _control. Genes were grouped by related function and the results are displayed using the Java Treeview program . A narrow color intensity scale (*yellow*, increased expression relative to control; *blue *decreased expression) is used to show the conditions under which changes in gene expression were first detectable. B) The data for highly expressed genes in panel A were plotted with a broader scale to better show the differences in gene expression. The complete dose-response and time-course analyses were performed twice with similar results and the data, presented as the ratio relative to control, are provided in Additional file 5.

To characterize how Zap1 target genes respond to a range of zinc availability, we performed additional microarray experiments in which we assayed their expression in wild type cells grown for 16 hours in batch cultures supplemented with various concentrations (i.e. 3, 10, 30, 100, or 300 μM) of zinc. RNA from cells grown in LZM + 3 mM ZnCl_2 _was used as the zinc-replete control for each of these comparisons and the data obtained are plotted in Fig. 4A (*left *panel). A narrow color scale was chosen for this panel to highlight the conditions under which detectable increases in gene expression occurred. Because this scale obscures the results obtained for the most highly induced genes (e.g. *ZRT1, YOR387C*), data for several genes are also plotted in Fig. 4B using a broader scale that allows for comparison of the expression levels across the full range of zinc conditions.  The numerical data from the experiments shown in Figure 4 are provided in Additional file 5.

From this dose-response analysis, it was apparent that Zap1 target genes show extremely varied responses to zinc deficiency. Some genes, such as *ZRT1*, *FET4*, *ZPS1*, and *YOR387C *responded to very mild conditions of zinc deficiency (i.e. LZM + 300 μM ZnCl_2_) while the *ZRT3 *and *IZH2 *genes were induced by moderate conditions of zinc deficiency (LZM + 100 or 30 μM ZnCl_2_). In contrast, most Zap1 target genes only responded to more severe deficiency conditions (LZM + 10 or 3 μM ZnCl_2_). Among these was *ADH4 *whose induction by low zinc correlated closely with the Zap1-mediated repression of the *ADH1 *and *ADH3 *genes. Also among the genes only responding to severe zinc limitation were *ZRG17 *and *TSA1*, i.e. genes likely to be involved in adaptation to zinc-limiting conditions (see Discussion).

Other patterns of expression were also observed. For example, induction of *ZRT2 *was observed in response to mild deficiency (LZM + 300 μM ZnCl_2_) and its repression by Zap1 was readily apparent in cells grown in LZM + 10 or 3 μM ZnCl_2_. No other Zap1 targets showed clear evidence for a similar combination of Zap1 induction and repression with the possible exceptions of *IZH2 *and *PEP4*. In contrast, several other genes (i.e. *MCD4, UBX6, ICY2, HNT1, URA10*) showed decreased expression under mild-to-moderate zinc deficiency but showed increased expression in severely zinc-limited cells. This profile may reflect the loss of activity of other transcription factors during moderate zinc deficiency that was compensated by up-regulation by Zap1 in severely zinc-limited cells.

Regulation of Zap1 target genes was also assessed over time in cells undergoing zinc withdrawal. Wild type cells were transferred from a zinc-replete medium (LZM + 1 mM ZnCl_2_) to a severely zinc-limiting condition (LZM + 1 μM ZnCl_2_) and gene expression was monitored over an 8-hour period. We predicted that genes that respond to mild zinc deficiency under the more steady-state conditions of batch culturing would also respond quickly to the mild zinc deficiency that would occur shortly after zinc withdrawal commenced. Consistent with this expectation, several genes induced by mild or moderate zinc deficiency (e.g. *ZRT1, ZPS1, YOR387C, ZRT3*) were induced shortly (i.e. within 1 hour) after transition from high to low zinc (Fig. 4A, *right *panel). Similarly, genes that responded only to severe deficiency in batch culture usually required much longer periods of zinc withdrawal (8 hours) for induction to be observed. Many of these genes decreased in expression level soon after transition to zinc-limiting conditions and, in some cases, were not increased relative to zinc-replete cells even after 8 hours in zinc-limiting medium. Induction was apparent after 16 hours in zinc-limiting conditions (dose-response analysis, Fig. 4A, *left *panel) suggesting that these genes require more than 8 hours of zinc withdrawal for their mRNA levels to increase.

The results in Fig. 4 indicated that there is generally a good correlation between the severity of the zinc deficiency required for induction of a Zap1 target gene under steady state conditions and its timing of induction following zinc withdrawal. However, some striking exceptions to this correlation were observed. For example, while *FET4 *and *ZRT2 *responded to mild zinc deficiency, they responded only slowly to zinc withdrawal. Conversely, while *ICY2 *responded only to severe zinc deficiency, it was highly induced within 30 minutes of zinc withdrawal. The underlying mechanisms and physiological significance of these intriguing differences in expression patterns are unclear.

### One possible mechanism underlying differential Zap1 target gene expression

**Figure 5:**
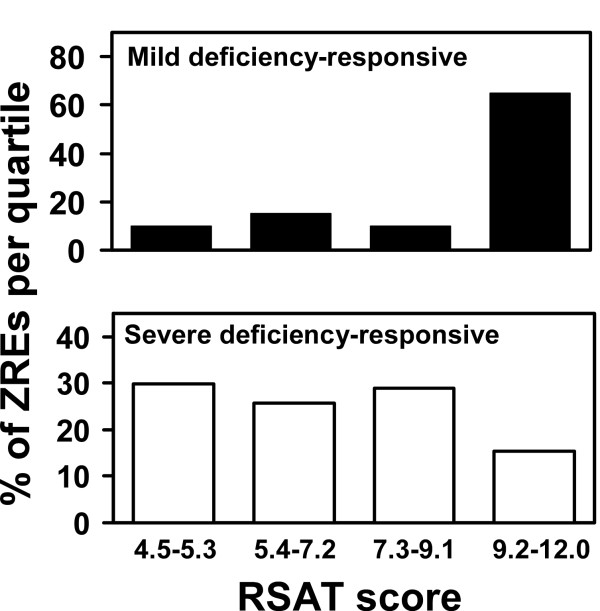
**Differences in ZREs among Zap1 target genes.** All ZREs from genes that respond to mild zinc deficiency and those that respond only to severe deficiency were divided into quartiles based on their RSAT scores. In the *upper *panel, the percentage of ZREs in each quartile for the genes responding to mild zinc deficiency are plotted. In the *lower *panel, the percentage of ZREs in each quartile for the genes responding to severe zinc deficiency are plotted. Chi-square analysis indicated that the different distributions among quartiles obtained with the two sets of ZREs are significant (P < 0.001).

The data in Figure 4 indicate that some Zap1 target genes respond to mild zinc deficiency while others respond only to severe deficiency. While many factors may contribute to these patterns of expression, one simple hypothesis is that the quality of ZREs within a gene's promoter determines its expression pattern. Specifically, genes with ZREs closely matching the Zap1 consensus sequence would respond to mild zinc deficiency because Zap1 could bind to those high affinity sites under those conditions when Zap1 levels are low. To activate promoters with weaker binding sites, the increased expression of Zap1 that occurs in zinc-limited cells would be required. This concept is similar to the differential binding of Zap1 to the ZREs found in the *ZRT2 *promoter [17]. Consistent with this hypothesis, we observed that the ZREs found in genes induced by mild deficiency (*ZRT1*, *ZRT2*, *ZPS1*, etc.) more closely match the consensus sequence (average RSAT score = 10.0) than do ZREs from genes that respond only to severe deficiency (average RSAT score = 7.1). This difference was even more striking when all of the ZREs were divided into quartiles based on RSAT score and the percentage of ZREs in each quartile were plotted. While ZREs among genes responding to only severe zinc limitation showed a fairly even distribution among quartiles, ZREs from genes responding to mild deficiency showed a clear bias toward the highest quartile (9.2–12.0) of RSAT scores (Fig. 5). Chi-square analysis confirmed that these are statistically significant differences (P < 0.001). No significant differences in the number of ZREs per promoter or the distances of the ZREs from the open reading frame were observed between the two sets of genes. Thus, we suggest that ZRE affinity may play a major role in dictating expression patterns observed among Zap1 target genes. It should be noted, however, that there are clear exceptions to this rule. For example, the *ZRG17 *promoter has a high affinity ZRE that exactly matches the consensus sequence but this gene only responds to severe deficiency. Other factors, such as chromatin structure, may influence binding of Zap1 to the *ZRG17 *promoter.

## Discussion

Zinc deficiency causes drastic changes in yeast gene expression. We previously reported that ~15% (934) of all yeast genes increased or decreased in expression in zinc-limited vs. zinc-replete yeast grown in batch cultures [3]. Similarly, De Nicola et al. reported that 381 genes were affected by zinc status in chemostat cultures [12]. Because zinc plays so many functional roles, the majority of these effects are likely to be indirect responses to changes in cellular processes resulting from decreased activity of key zinc-dependent proteins. To define the direct responses to zinc deficiency, we are identifying genes under the control of the Zap1 transcription factor. In this way, we can learn how cells respond specifically to the stress of zinc-limiting conditions.

We previously used a combination of microarray analysis and a motif identification algorithm to identify 46 potential Zap1 targets [3]. In this current study, we made important modifications to our previous approach by using alternative growth conditions for the microarray experiments and a different motif analysis algorithm that was better able to detect potential Zap1 binding sites. Using these new tools, we have extended many aspects of our previous work. First, this study provides additional confirmation for many genes previously proposed to be Zap1 targets (Table 1). For example, genes such as *MOH1*, *TKL2*, etc. were suggested to be direct Zap1 targets based on their Zap1-dependent induction in zinc-limited cells [3]. Their increased expression in cells expressing the Zap1^TC ^allele provides strong additional support for this hypothesis. In all, 33 of our original 46 potential targets were confirmed in this way. Several of the remaining 13 genes (e.g. *YBL048W*, *COS4*, *COS8, RAD27*) showed increased expression in Zap1^TC^-expressing cells although these effects did not meet our minimum cut-off value of 1.5-fold changes (Additional file 1).

In addition, we have added 31 potential new members to the Zap1 regulon. Sixteen of these genes were observed in our previous study to be zinc-responsive and Zap1-dependent [3] but were not considered to be direct Zap1 targets because we were unable to identify potential ZREs in their promoters using MEME (Table 2). With RSAT, we identified their potential ZREs and went on to show that many of these sequences were specifically bound by Zap1 *in vitro*. The response of these genes to the Zap1^TC ^allele also provides additional evidence for their direct regulation by Zap1. The other 15 new target genes responded to more severe zinc-limiting conditions than that used in our previous study (Table 3). These genes, plus four additional genes identified as Zap1 targets using other methods, i.e. *EKI1*, *PIS1, ZRR1*, and *ZRR2 *[18,21,22], brings the total of confirmed and potential Zap1 targets in the yeast genome to 81 genes.

**Figure 6:**
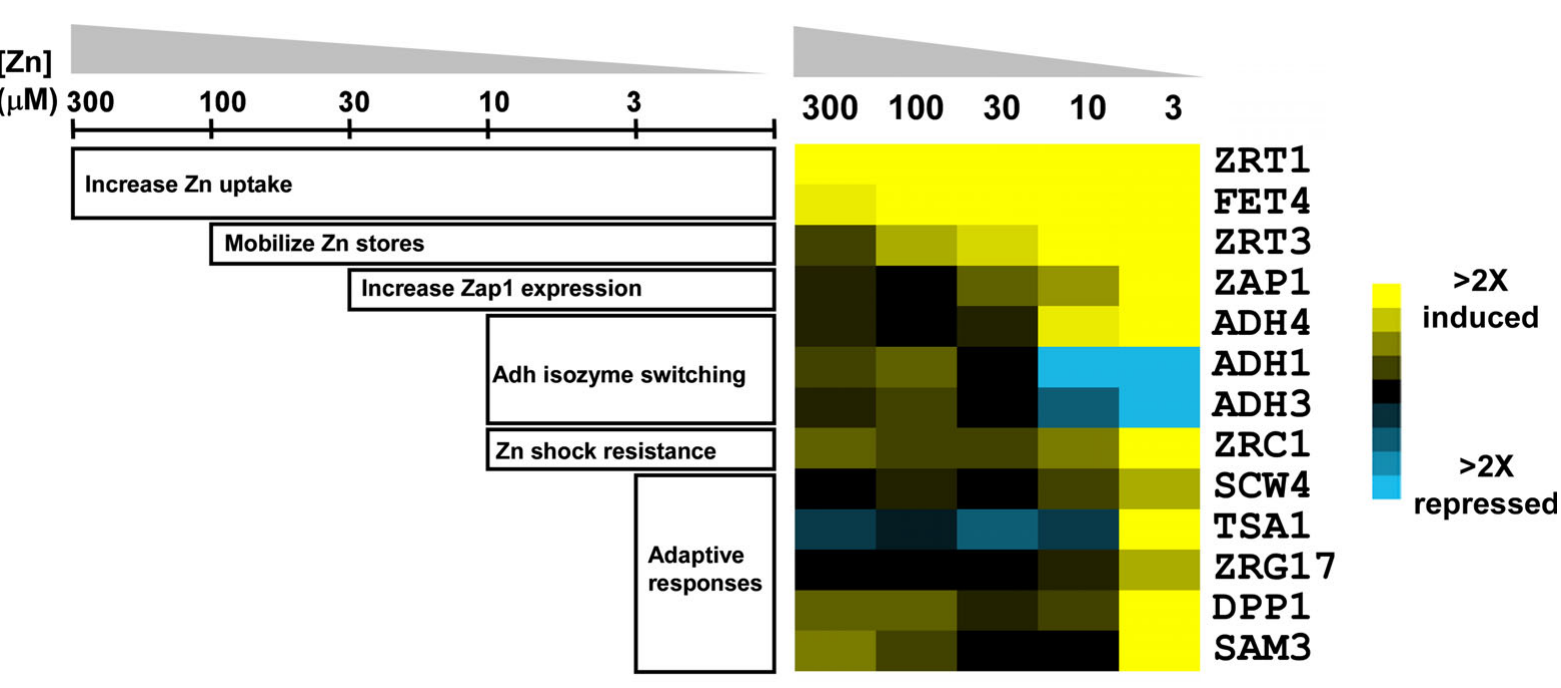
**The relationship between gene function and regulation by Zap1 in response to zinc status.** The top line indicates the range of zinc levels in our experiments ranging from mild (LZM + 300 μM ZnCl_2_) to severe (LZM + 3 μM ZnCl_2_) zinc deficiency. The bars below indicate the range of zinc over which each functional response occurs. Sample data from Figure 4A are included to illustrate the patterns of differential regulation for genes from different functional categories. See Table 4 for details regarding the various adaptive responses and the particular genes associated with both homeostatic and adaptive responses.

With the identification of many new targets of Zap1 activation, we are building a comprehensive picture of how yeast cells respond to zinc-limiting conditions. A summary of the functional roles of many of these genes is provided in Table 4 and a figure showing the general relationship of these genes to their response over a range of zinc levels is provided in Fig. 6. We can separate these responses conceptually into first- and second-lines of defense against the stress of zinc deficiency. The first-line defense genes play key roles in zinc homeostasis while many second-line defense genes allow the cells to adapt to zinc-limiting conditions when they can no longer obtain sufficient zinc for optimal growth. One caveat to this analysis is that we are assuming that changes in transcript levels are accompanied by similar changes in protein abundance and this assumption is not necessarily true. Nonetheless, the following discussion provides a clear and testable framework for understanding the cellular responses to zinc deficiency.

**Table 4:** Functional categories of genes regulated by the Zap1 transcriptional factor.

Functional Group	Zap1 Target Genes
First-line defenses: Zinc homeostasis	
Zinc uptake	*ZRT1, ZRT2, FET4, ZPS1*
Mobilize zinc stores	*ZRT3*
Increase transcription response	*ZAP1*
Zinc sparing	*ZRR1 (ADH1), ZRR2 (ADH3), ADH4*
Zinc shock resistance	*ZRC1*
Other zinc homeostasis genes	*IZH1, IZH2*
	
Second-line defenses: Adaptive responses	
	
Secretory pathway function	*ZRG17, MNT2, MCD4, YJR061W*
Cell wall function	*PST1, BAG7*, ***HSP12, SED1, SCW4, YOL155C, YIL169C, YJL171C***
Stress resistance	*GRE2, RAD27*, ***TSA1, CTT1, UTH1, HSP26***
Phospholipid metabolism	*DPP1, EKI1, PIS1, YJL132W*
Sulfur metabolism	***LAP3, SAM3, YPR003C***
Protein degradation	*PRC1, PEP4*, ***PRB1, ATG19, UBX6***
Carbohydrate metabolism	*NRG2, TKL2*, ***ENO1, ENO2, TDH1***
Purine/pyrimidine metabolism	*URA10, ADE17*, ***HNT1***
Mitochondrial function	***FMP43, PET20, PET10***
	
Other	*VEL1, MOH1, GPG1, COS6, ICY2, COS1, PHM7, **PIG2, TIS11, ALD3, PUT4**, COS2, ZIP1, COS4, COS8, TPO5, COS3, YNL254C, YOR387C, YLL020C, YOL131W, **YBL029W, YHR214W-A, YMR181C, YLL053C **, YBL048C, YMR086W, YNL234W*

Genes shown in bold are new targets in this study

Among the first-line defense genes, *ZRT1*, *ZRT2*, and *FET4 *are induced by mild zinc deficiency to increase the ability of the cell to accumulate zinc from its environment. *ZPS1 *is also induced under these mild conditions and we have previously proposed that its product, a secreted protein related to metalloproteases, may also be involved in zinc acquisition by degrading extracellular proteins and releasing any bound metals [3]. Similarly, *ZRT3 *is up-regulated by mild zinc deficiency and also responds rapidly to zinc withdrawal. Thus, mobilization of zinc stores from the vacuole is also a first-line response. Induction of the *YOR387C *gene in response to mild zinc deficiency suggests that this gene is also involved in the first-line defense against zinc limitation. The function of this protein is not yet known but its pattern of regulation by zinc suggests that it may play a role in zinc uptake or vacuolar zinc export. *TIS11 (*also known as *CTH2*) is also induced in LZM + 300 μM ZnCl_2_. Tis11 binds to specific mRNAs and signals their degradation in response to iron limitation [25]; its role in zinc deficiency is not yet determined.

Other first-line defenses to zinc deficiency include an increase in *ZAP1 *expression mediated by Zap1 autoregulation [1]. The resulting increase in Zap1 protein level may maximize target gene expression and is also likely to be responsible for repression of *ZRT2 *under these conditions [17]. In support of this latter hypothesis, we note that *ZRT2 *repression correlates well with the observed increase in *ZAP1 *mRNA levels. In addition, the coordinated switch from *ADH1/ADH3 *expression to *ADH4 *expression likely represents a mechanism to conserve zinc for other uses [18]. Finally, we observed an increase in *ZRC1 *expression in moderate-to-severely zinc-deficient cells. We showed previously that this induction is required for cells to survive the stress of zinc shock, i.e. when zinc-deficient cells are re-supplied with zinc [26]. The high activity of zinc uptake transporters in these cells results in rapid zinc overload and the increased activity of Zrc1 is needed to sequester the excess zinc in the vacuole.

Induction of many Zap1 target genes occurred only in response to the most severe zinc-limiting condition we tested (LZM + 3 μM ZnCl_2_) and only slowly in response to zinc withdrawal. We consider these genes to be the second line of defense against zinc deficiency. Most of these second-line defense genes appear to be involved in the adaptation to zinc deficiency rather than playing a role in zinc homeostasis (see below). This transition from homeostatic to adaptive mechanisms correlates well with what we know about cell growth under these conditions. We previously showed that cells grow at their maximum growth rate in LZM supplemented with ≥ 10 μM ZnCl_2 _but their growth rate decreases in media containing less zinc [14]. Thus, under conditions that induce the second-line defense genes, zinc homeostatic mechanisms are no longer capable of supplying sufficient zinc for optimal function of cellular processes. Our results indicate that it is under these conditions of suboptimal zinc that adaptive responses occur.

One important functional category of second-line defense involves secretory pathway function (Table 4). The *ZRG17 *zinc transporter gene is up-regulated presumably to maintain the zinc status of the endoplasmic reticulum [27,28]. *MCD4 *and *MNT2 *encode enzymes required for protein modifications (GPI anchor synthesis and O-linked glycosylation), that occur in the ER and the Golgi, respectively [29,30]. *YJR061W *encodes an ER-localized protein [31] related to Mnn4, which is involved in both N- and O-linked glycosylation [32]. Increased expression of these proteins may help maintain the efficiency of these processes under the adverse conditions of severe zinc deficiency. Consistent with this hypothesis, Mcd4 is thought to be a zinc-dependent enzyme [33].

Another group of second-line defense genes are involved in cell wall function (Table 4). These include *PST1*, *HSP12*, *SED1*, and *SCW4*. While their specific functions are unclear, these proteins all reside in the cell wall suggesting that cell wall remodeling occurs under zinc-limiting conditions. These alterations may be important to maintain the structural integrity of the cell wall. Consistent with this hypothesis, disruption of zinc transport into the secretory pathway caused increased sensitivity to an inhibitor of cell wall function, calcofluor white [34]. This observation suggests that defects in cell wall synthesis occur in zinc-limited cells.

A third functional category of second-line defense genes are involved in stress resistance (Table 4). Our results implicate *TSA1*, *CTT1*, *UTH1*, and *HSP26 *as Zap1 targets along with previously identified targets *RAD27 *and *GRE2*. *RAD27 *encodes a nuclease that is required for the stability of minisatellite repeats in genomic DNA [35,36]. The recent observation that zinc deficiency in yeast destabilizes these DNA repeats [37] is consistent with a role of Rad27 in promoting genome stability in zinc-limited cells. *GRE2 *encodes a methylglyoxal reductase [38]. Methylglyoxal is a byproduct of glycolysis that can react with proteins to disrupt their function [39]. We note with interest that two major targets of damage by methylglyoxal, the enolases encoded by *ENO1 *and *ENO2 *[39], are also up-regulated by Zap1 (Table 3). *TSA1*, *CTT1*, and *UTH1 *are involved in resistance of the cell to oxidative stress. We recently showed that yeast cells grown under severe zinc-limiting conditions experience increased oxidative stress [23]. In addition, we demonstrated that induction of *TSA1*, encoding the major cytosolic peroxiredoxin, in low zinc is required to resist that increased stress. *CTT1*, encoding the cytosolic form of catalase [40], may be up-regulated for similar reasons. Uth1 is a mitochondrial protein of unknown function that is required for superoxide resistance [41] so its induction by Zap1 may also be related to oxidative stress resistance. Lastly, Hsp26 is a member of the small heat shock protein family that has chaperone activity and protects proteins from misfolding [42]. The regulation of these genes by Zap1 may help temper the various stresses experienced by zinc-limited cells. Other functional categories of Zap1 target genes induced by severe zinc deficiency include phospholipid synthesis, protein degradation, and carbohydrate, sulfur, and purine/pyrimidine metabolism (Table 4). On a final note, more than half of the genes now identified as potential Zap1 targets (47 of 81) have mammalian orthologs. Regulating the expression of these genes may also be important for cell growth under low zinc conditions in mammals.

## Conclusion

In this report, we have identified 31 new potential targets of Zap1 and have characterized the response of these and previously identified target genes to changes in zinc status and to time following zinc withdrawal. This analysis of Zap1 transcriptional regulation is providing unexpected and exciting new insights into the mechanisms of metal nutrient homeostasis in yeast and is telling us much about how these cells adapt to growth under the stress of zinc deficiency. Future studies will address the detailed roles of these various adaptive responses to cell growth under zinc-limiting conditions.

## Methods

### Growth conditions and strains

Yeast cells were grown in YPD (YP medium + 2% glucose) and in synthetic defined SD medium with 2% glucose or 2% galactose and any necessary auxotrophic requirements. YPD and SD are zinc-replete media because they contain micromolar levels of zinc and lack strong zinc chelators. Yeast were made zinc limited by culturing in low zinc medium (LZM) prepared as previously described [43]. LZM is zinc limiting because it contains 1 mM EDTA and 20 mM citrate to buffer metal availability. Therefore, only a small fraction of the total zinc in LZM medium is available for uptake by cells. Zinc was added to LZM as ZnCl_2_. The wild type strain DY1457 (*Matα ade6 can1 his3 leu2 trp1 ura3*) was used in all experiments.

### Microarray analysis

The data for Experiment 1 (E1) and Experiment 2 (E2) are from Lyons et al. [3]. The new microarray analyses used cells grown under two different paired conditions and each experiment was performed in duplicate with independent cultures. In Experiment 3 (E3), wild type (DY1457) cells were transformed with the vector (pYef2) or a plasmid (pYef2-Zap1^TC^) encoding a constitutive allele of Zap1 under the regulation of the galactose-inducible *GAL1 *promoter. The plasmid pYef2 and pYef2-Zap1^TC ^constructs were previously described [7]. These transformants were inoculated into zinc-replete SD medium + 2% galactose + 1 μM ZnCl_2 _and grown for 20–24 h before harvesting at an optical density measured at 600 nm (OD_600_) of ~0.8. In Experiment 4 (E4), wild type (DY1457) cells were grown in a severely zinc-limiting medium (LZM + 3 μM ZnCl_2_) and in a zinc-replete medium (LZM + 3 mM ZnCl_2_) for 14–16 hours and harvested at an OD_600 _of ~0.7. For the dose-response analysis, wild type (DY1457) cells were grown in LZM + 3 mM ZnCl_2_, or LZM + 300, 100, 30, 10 or 3 μM ZnCl_2_. Transcript levels were assayed using microarrays in which each sample was paired with the zinc-replete (LZM + 3 mM ZnCl_2_) control. For the time-course studies, wild type (DY1457) cells were grown to exponential phase in a zinc-replete medium (LZM + 1 mM ZnCl_2_), washed twice in LZM with no added zinc, and then transferred to a zinc-limiting medium (LZM + 1 μM ZnCl_2_) for 8 hours. Transcript levels were assayed using microarrays in which each sample was paired with the zinc-replete T_o _control. These experiments were also performed in duplicate with independent cultures. Total RNA was extracted from cells grown as described above with hot phenol and mRNA was isolated from total RNA using the PolyATtract mRNA Isolation System IV kit (Promega). Cy3-dUTP or Cy5-dUTP was incorporated during reverse transcription of the polyadenylated RNA [44]. The fluorescently labeled products were recovered and hybridized to yeast whole-genome microarrays, washed, and scanned as previously described [44].

To remove intensity-dependent measurement artifacts, we normalized the log Cy3/Cy5 fold changes within each microarray slide using the locally weighted scatterplot smoothing (LOWESS) algorithm [45]. Genes exhibiting sufficiently high fold change were screened for subsequent analysis. We chose an arbitrary cut-off value of a fold change ≥ 1.5 based on the average of two independent microarrays with the provision that both arrays showed a fold change of at least 1.4. One gene, *ENO2*, did not fully satisfy these criteria but was selected for further analysis because of the presence of a potential ZRE in its promoter. Subsequent S1 nuclease protection assays confirmed the zinc- and Zap1-responsive regulation of *ENO2*.

### Promoter motif and ZRE distribution analysis

The ZREs in the promoters of the 46 potential Zap1 targets previously identified with the Multiple Expectation Maximization for Motif Elicitation (MEME) program (please see Availability & requirements for more information) [46] were used to generate a position-specific probability matrix with Regulatory Sequence Analysis Tools (RSAT – please see Availability & requirements for more information)[47]. Potential ZREs in the promoters (nucleotide positions -1000 to +1 where +1 is the first base of the ATG start codon) of zinc- and Zap1-responsive genes were then identified using this matrix and RSAT. To assess the distribution of potential ZREs in promoter regions, PatMatch (please see Availability & requirements for more information) was used to identify ZRE-like sequences in the promoters of other yeast genes using the input sequence of ACCYKNRRKGT (Y = C or T, K = G or T, R = A or G, N = any base). 138 genes were identified that contain one or more copies of this sequence in their promoters but were not zinc- and Zap1-responsive; 156 total ZRE-like sequences were found in these promoters. The randomness of distribution of these sequences was assessed using the chi-square test. WebLogo (please see Availability & requirements for more information) [48] was used to generate the graphical representation of the ZRE consensus sequence shown in Figure 1B. To determine if the RSAT scores of ZREs in promoters that respond to mild deficiency were significantly different from those in promoters that respond only to severe deficiency, the total number of ZREs were divided into equal quartiles and then the ZREs in the two sets were divided among those quartiles. The chi-square test was then used to determine if the distribution of scores between those sets was statistically significant.

### RNA analysis

S1 nuclease protection assays were performed with total RNA as described [49]. The oligonucleotide probes used for these experiments are described in Additional file 3. For each reaction, 15 μg of total RNA was hybridized to ^32^P-end-labeled DNA oligonucleotide probes before digestion with S1 nuclease and separation on a 10% polyacrylamide, 5 M urea polyacrylamide gel. Band intensities were quantitated by PhosphorImager analysis (PerkinElmer, Inc.).

### Electrophoretic mobility shift assays

The Zap1 DNA binding domain (Zap1_DBD_, residues 687–880) was expressed in *E. coli *as a fusion to glutathione *S*-transferase, purified, and then the glutathione *S-*transferase tag was removed with thrombin as previously described [5]. Electrophoretic mobility shift assays were performed as previously described using purified Zap1_DBD _protein and radiolabeled ZRE oligonucleotides (Additional file 4). Briefly, 15 μl reactions were prepared containing 0.5 pmol of radiolabeled DNA oligonucleotide (20,000 cpm/pmol), 10 mM Tris-HCl (pH 8.0), 10 mM MgCl_2_, 50 mM KCl, 1 mM DTT, 0.02 mg/ml poly(dI-dC), 0.2 mg/ml bovine serum albumin, 0.04% NP-40, 10% glycerol, and the indicated concentrations of purified Zap1_DBD_. After incubation for 1 hour at room temperature, the samples were resolved on 6% polyacrylamide gels. Gels were dried onto blotting paper, and the signals were visualized by autoradiography.

### Microarray data

All microarray data can be downloaded from the Gene Expression Omnibus database under accession number GSE11983.

## Abbreviations

ZRE- Zinc-responsive domain, MEME- Multiple Expectation Maximization for Motif Elicitation, RSAT- Regulatory Sequence Analysis Tools, LZM- low zinc medium, SD- synthetic defined medium, DBD- DNA binding domain

## Availability and requirements

Multiple Expectation Maximization for Motif Elicitation (MEME) program:  

Regulatory Sequence Analysis Tools (RSAT): 

PatMatch: 

WebLogo: 

## Authors' contributions

C–YW performed the experiments and was the main author of the manuscript. AJB assisted in the microarray experiments and in the data analysis. LMC and MAN assisted in the statistical analysis of the array results. DRW provided critical intellectual contributions and assisted in writing the manuscript. DJE participated in experimental design and interpretation and assisted in writing the manuscript. All authors read and approved the final manuscript.

## Supplementary Material

Additional file 1 Microarray results of Zap1 target genes from Lyons et al. (ref. [3]) not confirmed by Experiment E3. Click here for file

Additional file 2 Microarray results for potential Zap1 targets identified in this study that lack detectable ZREs. Click here for file

Additional file 5 Microarray results for Zap1 targets from time course and dose response studies. Click here for file

Additional file 3 Oligonucleotides used for S1 nuclease protection assays. Click here for file

Additional file 4 Oligonucleotides used for electrophoretic mobility shift experiments. Click here for file
