# Supplementary material for: Differential control of Zap1-regulated genes in response to zinc deficiency in Saccharomyces cerevisiae
Source: BMC Genomics. 2008 Aug 1;9:370. doi: 10.1186/1471-2164-9-370 (PMC2535606; doi:10.1186/1471-2164-9-370)
Supplement: Additional file 1 — Microarray results of Zap1 target genes from Lyons et al. (ref. [3]) not confirmed by Experiment E3. [file 1471-2164-9-370-S1.pdf]

Additional file 1. Zap1 target genes from Lyons et al. not confirmed by E3.

ORF	Gene	Function	Fold induction				ZRE		
			E1[a]	E2[a]	E3-1[b]	E3-2[b]	start[c]	score[d]	sequence
YBL048W		function unknown	7.0	2.0	1.5	nd	-858	8.7	CCCTTGAGGGA
YBR066C	*NRG2*	transcription factor that mediates glucose repression	5.1	2.2	1.0	0.9	-920	7.8	AACTTGAGGGT
YBR302C	*COS2*	vacuole protein, function unknown	2.1	2.0	nd	nd	-592	5.3	AACCTAGAGGT
							-314	8.9	ACCCTAAATGT
							-217	5.0	ACCGGAAATGT
YDR285W	*ZIP1*	synaptonemal complex protein, meiotic recombination	4.5	2.1	1.1	1.1	-575	9.6	ACCTGAAAGGT
YFL062W	*COS4*	vacuole protein, function unknown	2.6	2.0	1.3	1.6	-865	5.0	AACTTAAATGT
							-584	5.6	AACTTAGAGGT
							-309	9.1	ACCTTAAATGT
YHL048W	*COS8*	ER protein, function unknown	2.2	2.1	1.4	1.4	-865	5.0	AACTTAAATGT
							-586	5.3	AACCTAGAGGT
							-314	9.1	ACCTTAAATGT
YKL113C	*RAD27*	nuclease, DNA replication and repair	2.2	4.7	1.3	1.2	-997	10.2	ACCCTCCGGGT
							-342	5.4	ACCAGCCGGGT
YKL174C	*TPO5*	putative polyamine transporter	3.2	2.0	0.9	1.1	-493	8.4	ACCATAAGGGT
YML132W	*COS3*	enhancer of Na+/H+ antiporter function	2.7	2.2	1.3	nd	-592	5.3	AACCTAGAGGT
							-314	8.9	ACCCTAAATGT
YMR086W		ribosome-associated protein	1.9	2.4	1.5	1.4	-565	7.4	ACCTCGAGTGT
							-145	9.7	ACCTTAAAGGA
YNL234W		similar to globins, function unknown	2.6	2.1	1.2	0.9	-68	7.2	CCCTTGAGGGG
YOL151W	*GRE2*	methylglyoxal reductase	2.5	2.6	1.0	1.0	-920	9.8	GCCTTGAGGGT
YPL154C	*PEP4*	vacuolar proteinase A	2.6	2.1	1.0	1.1	-396	8.2	GCCTTCCGGGT

a) Expression ratios are the average of two independent microarray experiments (Lyons et. al. 2000).

b) Results from two independent microarray experiments (E3-1 and E3-2) are shown.

c) Numbers indicate the distance from the ATG initiation codon.

d) Score calculated for each sequence with a position-specific scoring matrix generated by RSAT.
