# Supplementary material for: Differential control of Zap1-regulated genes in response to zinc deficiency in Saccharomyces cerevisiae
Source: BMC Genomics. 2008 Aug 1;9:370. doi: 10.1186/1471-2164-9-370 (PMC2535606; doi:10.1186/1471-2164-9-370)
Supplement: Additional file 2 — Microarray results for potential Zap1 targets identified in this study that lack detectable ZREs. [file 1471-2164-9-370-S2.pdf]

**Additional file 2. Potential Zap1 targets lacking detectable ZREs.**

| ORF | Gene | Function | Fold induction | | | |
|---|---|---|---|---|---|---|
| | | | E1[a] | E2[a] | E3-1[b] | E3-2[b] |
| YBR285W | | function unknown | 6.7 | 2.8 | 3.9 | 3 |
| YKL163W | *PIR3* | cell wall protein | 5.9 | 2.4 | 3.5 | 3.3 |
| YMR170C | *ALD2* | aldehyde dehydrogenase, Coenzyme A synthesis | 11.5 | 4.7 | 1.7 | 1.7 |
| YNR066C | | membrane protein, function unknown | 3.7 | 2.1 | 1.7 | 1.4 |

a) Expression ratios are the average of two independent microarray experiments (Lyons et. al. 2000).

b) Results from two independent microarray experiments (E3-1 and E3-2) are shown.
