# Supplementary material for: Differential control of Zap1-regulated genes in response to zinc deficiency in Saccharomyces cerevisiae
Source: BMC Genomics. 2008 Aug 1;9:370. doi: 10.1186/1471-2164-9-370 (PMC2535606; doi:10.1186/1471-2164-9-370)
Supplement: Additional file 3 — Oligonucleotides used for S1 nuclease protection assays. [file 1471-2164-9-370-S3.pdf]

Additional file 3. Oligonucleotides used for S1 nuclease protection assays.

Target Gene	Oligonucleotide
ZRT1	5′-GGCCACACAGATTGGTGTGGTTAACCCATACGCAACACATAGGGCC CATGGCCACCTGATGCCA-3′
CMD1	5′-GGGCAAAGGCTTCTTTGAATTCAGCAATTTGTTCTTCGGTGGAGCC-3′
TKL2	5′-GCCAATCCTAGTGGTGCACCTGGGTGGCCAGATTGCATAT-3′
PST1	5′-GGCCGTGGCATGTGAGCTTATGGTACAGGAAGAGGGCGCAT-3′
YJL132W	5′-GCACCTCTTGTTAAATAAAATAGATGGGTAGTAACCTTGA-3′
ICY2	5'-CGGCATAAGTCGATCTGTCCGGGCTATGCGAGAGCGCGAA-3'
HSP26	5′-GCGTAGCCTCTTAAGCCGCCTTCACCCAGCAATCTACCGGG-3′
SED1	5′-GGAGATGGAAGAGGAGGAAGTGACATCGGTGGAAGAGATGA-3′
CTT1	5′-GGGCCGTCTGGTCTTGAGTATTGAGAAGCGTATGGACAGC-3′
UBX6	5′-GCCAAGAATTGTCCCCGGCTGCTGAACTAATGTAAGTAGT-3′
TIS11	5′-GGTACTGGTAGTCATTTAATGGATTTTGCTCTTGATCCACAA-3′
LAP3	5′-GCCAATTGATGGGTTAAGTCGGATTGAAACTCTTTACCTT-3′
YOL155C	5′-CCGGAGACTGATGAGCCCGATTCAGTGGCGGAGGATGAACTAAGA-3′
HNT1	5'-GCATACGAGTACTTTGTTTCAATCAATTTGAAGGACAAGG-3'
PRB1	5'-CGTGGTGGTCGGCGGCATTTTCAAGATTTGGGATGGTTGG-3'
SCW4	5′-GCGGGAGCAGCAAGAGTAGCAGCAGATAAAAGAGAGGCAGGGATG-3′
ENO2	5'-CCCATCCACTTGGATTTGTCTTCATCTCTCATTTCCGGGAT-3'
YIL169C	5′-TACCACAGAGGTGGATGAGCTGTTGCTTGAAATTGAGGTGTCGCCA-3′
UTH1	5′-GCGCCGTCTGCGTTGACGTACTGAGTAACGGTGACGGTAA-3′
SAM3	5'-GGAATCCTTCATTCTTCTGATCAGTGAACCAGATCCGCTT-3'
