# Supplementary material for: Differential control of Zap1-regulated genes in response to zinc deficiency in Saccharomyces cerevisiae
Source: BMC Genomics. 2008 Aug 1;9:370. doi: 10.1186/1471-2164-9-370 (PMC2535606; doi:10.1186/1471-2164-9-370)
Supplement: Additional file 4 — Oligonucleotides used for electrophoretic mobility shift experiments. [file 1471-2164-9-370-S4.pdf]

Additional file 4. Oligonucleotides used for EMSA experiments.

ZRE[a]	Oligonucleotide[b]
TSA1 ZRE1	5′-ggccCTGTTCTGGCCCGTCGGGTTTTCTGACAAA-3′<br>3′-GACAAGACCGGGCAGCCCAAAAGACTGTTTagct-5′
TSA1 mZRE1	5′-ggccCTGTTCTGTAAATGATTTGTTTTCTGACAAA-3′<br>3′-GACAAGACATTTACTAAACAAAGACTGTTTagct-5′
HSP26 ZRE	5'-ggccCCTAAAGAACCTTGCCTGTCAAGGTGCATT-3′<br>3′-GGATTTCTTGGAACGGACAGTTCCACGTAAagct-5′
SED1 ZRE	5'-ggccACCCATTACCCTTATAGGATTAATGTAAGC-3'<br>3′-TGGGTAATGGGAATATCCTAATTACATTCGagct-5′
UBX6 ZRE1	5'-ggccCAGGAGGAACCTTAATGGATTTACCTCTAG-3'<br>3′-GTCCTCCTTGGAATTACCTAAATGGAGATCagct-5′
TIS11 ZRE1	5'-ggccGCCTTCCAGCCCAGAGGGTTCAAACGTTAT-3′<br>3′-CGGAAGGTCGGGTCTCCCAAGTTTGCAATAagct-5′
YOL155C ZRE	5'-ggccAGACCACGTCCTGCACGGTTTTGCAGACTG-3'<br>3′-TCTGGTGCAGGACGTGCCAAAACGTCTGACagct-5′
HNT1 ZRE	5'-ggccGCCACGTAGCCTCAAAGGTTGAATTGACAC-3'<br>3′-CGGTGCATCGGAGTTTCCAACTTAACTGTGagct-5′
PRB1 ZRE	5'-ggccGACAATCAACCCTCATGGCGCCTCCAACCA-3′<br>3′-CTGTTAGTTGGGAGTACCGCGGAGGTTGGTagct-5′
ENO2 ZRE	5'-ggccAAGTGTCGACGCTGCGGGTATAGAAAGGGT-3'<br>3′-TTCACAGCTGCGACGCCCATATCTTTCCCAagct-5′
SAM3 ZRE	5'-ggccTTTCTTTGTCCCCTGCGGTTGCATAGACAT-3'<br>3′-AAAGAAACAGGGGACGCCAACGTATCTGTAagct-5′

[a] *TSA1* mZRE1 is mutated such that each position in the *TSA1* ZRE1 was altered by a transversion mutation.

[b] The potential ZREs (or the mutated region in *TSA1* mZRE1) in each complementary oligonucleotide pair are indicated by the line. The lower case letters indicate *Eag*I- and *Sal*I-complementary overhangs included for cloning these fragments into a *lacZ* reporter plasmid for future studies.
